# Serum Chitinase 3-like-1 (CHI3L1) Has Good Correlation with Fecal Calprotectin Levels in Pregnant IBD Patients

**DOI:** 10.3390/jpm16030145

**Published:** 2026-03-03

**Authors:** Hagai Schweistein, Rachel Gingold-Belfer, Adi Rave, Ahinoam Glusman-Bendersky, Hadar Amir-Barak, Jacob E. Ollech, Ariella Bar-Gil Shitrit

**Affiliations:** 1 Division of Gastroenterology, Meir Medical Center, Kfar Saba 4428164, Israel; 2 Gray Faculty of Medical and Health Sciences, Gray School of Medicine, Tel-Aviv University, Tel-Aviv 69978, Israel; 3 Division of Gastroenterology, Rabin Medical Center, Beilinson Hospital, Petach Tikva 49100, Israel; 4 Clalit Health Services, Sharon Shomron 4428164, Israel; 5 IBD MOM Unit, Digestive Diseases Institute, Eisenberg R&D Authority, Shaare Zedek Medical Center, Jerusalem 9103102, Israel; 6 Faculty of Medicine, Hebrew University of Jerusalem, Jerusalem 9112102, Israel

**Keywords:** chitinase-3-like-1 (CHI3L1), inflammatory biomarker, ulcerative colitis, Crohn’s disease, pregnancy, personalized treatment

## Abstract

**Background**: Monitoring inflammatory bowel disease (IBD) during pregnancy is challenging due tolimited use of invasive tools. While fecal calprotectin is considered reliable, its use is limited by patient adherence and availability. Blood-based markers, such as serum chitinase-3-like-1 (CHI3L1), offer a promising alternative. **Aim**: To evaluate whether serum CHI3L1 reflects disease activity in pregnant IBD patients, compared to standard markers and clinical questionnaires. **Methods**: Pregnant IBD patients were recruited from a multidisciplinary clinic. Blood samples were collected to assess inflammatory markers. Stool samples were used to measure calprotectin levels. Each visit was classified as a distinct sample for analysis. Correlations between CHI3L1 and disease activity markers were examined. **Results**: A total of 124 samples from 80 pregnant IBD patients were analyzed: 90 from Crohn’s disease (CD) patients and 34 from ulcerative colitis (UC) patients. CHI3L1 levels showed a significant positive correlation with fecal calprotectin (r_p_ = 0.366, *p* = 0.008), ESR (r_p_ = 0.358, *p* = 0.001), CRP (r_p_ = 0.478, *p* < 0.001) and standardized clinical scoring questionnaires. Elevated CHI3L1 (>56.6 ng/mL) is a risk factor for active disease (OR 8.78, 95% CI 1.54–49.83, *p* = 0.014). **Conclusions**: Serum CHI3L1 is positively associated with established markers of inflammation and may serve as a useful non-invasive biomarker for monitoring IBD activity during pregnancy, a medical condition in which invasive procedures are not recommended. Based on CHI3L1 levels, personalized treatment for pregnant IBD patients can be tailored. However, further validation is recommended.

## 1. Background

Monitoring inflammatory bowel disease (IBD) activity during pregnancy continues to be a challenge due to the desire to avoid invasive diagnostic methods as well as the limitations of the use of non-invasive markers. Although fecal calprotectin is considered a reliable and widely used marker [1,2,3], its practical utility can sometimes be limited by patients’ reluctance to or difficulty in providing stool samples and by logistical challenges related to sample collection [4,5]. Blood-based markers may offer a more convenient alternative in some settings, as they are generally easier to obtain and integrate into routine clinical practice, potentially improving patient adherence. These advantages facilitate their integration into clinical practice and improve the likelihood of patient adherence. Pregnancy is a condition in which inflammatory markers such as erythrocyte sedimentation rate (ESR) and C-reactive protein (CRP) increase regardless of the presence of pathological inflammation due to physiological and immunological changes [6,7,8]. As there are no better alternatives, these inflammatory markers are used in the clinic [9]. Chitin, one of the most common biopolymers in nature, can be found in many living organisms, including insects, fungi, crustaceans, and other marine animals [10]. Chitin is broken down in nature by chitinases previously unknown in mammals. However, it has been discovered that chitinases, as well as structurally similar chitinase-like proteins (CLPs), including glycoprotein chitinase 3-like-1 (CHI3L1), are also found in mammals [11] and have a role in activating inflammatory responses [12]. Previous research has shown that CHI3L1 is increased in inflammatory processes, which are mediated by the Th2 response, such as asthma and allergic reactions [13,14]. The CHI3L1 protein has been established as an indicator of inflammation and cancer in various non-gestational conditions, including in IBD [15]. Multiple studies provide a basis for assuming that serum CHI3L1 does not increase during normal pregnancy in a healthy woman [16,17]. However, the study of CHI3L1 levels in pregnant women with IBD has not yet been investigated. Assuming the level of this protein does not change during a normal pregnancy, it has been hypothesized that abnormal blood levels of CHI3L1 could serve as an effective inflammatory marker for the activity of inflammatory bowel disease during pregnancy. The aim of this study was to investigate whether serum CHI3L1 levels can serve as a reliable marker to indicate disease activity in pregnant IBD patients. Thus, we compared CHI3L1 levels to established inflammatory markers, including calprotectin, CRP, and ESR, and examined the correlation between serum CHI3L1 levels and the clinical status of pregnant women with IBD, as assessed by standardized clinical scoring questionnaires.

## 2. Patients and Methods

Study Population: We recruited pregnant women diagnosed with IBD, including Crohn’s disease (CD) and Ulcerative colitis (UC), who were followed at Shaare Zedek Medical Center (SZMC). Each visit was categorized as a separate data point for analysis. All participants provided written informed consent, approved by the hospital’s Institutional Review Board (IRB) in accordance with the Helsinki Declaration.

Inclusion Criteria:

# Provision of written informed consent.

# Age ≥ 18 years.

# Pregnant women diagnosed with CD orUC for at least six months based on imaging, endoscopy, or pathology findings and under follow-up at the SZMC.

Exclusion Criteria:

# Inability to provide informed consent.

# The presence of other inflammatory or autoimmune diseases, such as: cancer, rheumatoid arthritis, osteoarthritis, ankylosing spondylitis, hepatic fibrosis, severe purulent meningitis, pneumonia, or asthma.

# Use of corticosteroids >20 mg per day.

# Use of anti-allergic medications.

Clinical Questionnaires: Clinical questionnaires were used to assess disease activity among patients. Participants completed validated disease activity assessment questionnaires approximately one week prior to clinic visit. Disease activity was assessed using the Crohn’s Disease Activity Index (CDAI) for CD [18] or the Partial Mayo score for UC [19]. All participants in the study completed the disease-specific clinical questionnaires. Active disease was defined as a CDAI ≥ 220 and a partial Mayo score ≥ 5.

Blood Sampling, Serum Storage, and Data Collection: During clinic visits, participants underwent blood sampling to measure serum levels of Chitinase 3-like-1 (CHI3L1), CRP and ESR. Stool samples for calprotectin level analysis were collected on the day of the clinic visit and sent to the central laboratories of the hospital for processing. During the clinic visit, approximately 10 mL of blood was collected into serum separator tubes. After a 30 min rest period, the tubes were centrifuged at 2500 rpm for 15 min at 21 °C. The serum was separated and aliquoted into Eppendorf tubes for storage at −80 °C until analysis. Samples were anonymized and coded to ensure confidentiality during laboratory testing.

CHI3L1 Measurement: According to the manufacturer’s instructions, serum CHI3L1 levels were measured using an ELISA assay kit (Quantikine ELISA #DC3L10, R&D Systems, Minneapolis, MN, USA). Results were reported in ng/mL. Assay calibration was validated using previously tested serum samples from our laboratory. According to the manufacturer’s reference data for the CHI3L1 assay, the mean serum concentration was 36.0 ng/mL with a standard deviation of 20.6 ng/mL. Therefore, values exceeding one standard deviation above the mean (>56.6 ng/mL) were defined as elevated.

CRP and ESR Measurements: Serum CRP and ESR levels were analyzed at the central laboratories of SZMC using standard methods. Relevant clinical and demographic data were collected during clinic visits. Fecal Calprotectin Measurements: Fecal calprotectin levels were analyzed at the central laboratories of SZMC using an enzyme-linked immunoassay.

Statistical Analysis: Quantitative variables were expressed as means ± standard deviation. Categorical variables were expressed by frequencies. Univariate analysis was used to compare baseline characteristics; continuous normally distributed variables were compared by independent T-test, and those abnormally distributed were compared by Mann–Whitney test. Categorical variables were compared by Chi-Square test (or Exact Fisher test, as appropriate). Correlation analysis was performed; linear correlations were assessed using Pearson, while non-linear correlations were assessed using Spearman. Multivariate analysis using logistic regression was performed.

Statistical significance was set at *p* < 0.05. Statistical analysis was performed using the SPSS software (version 29).

## 3. Results

Study population: A total of 124 samples were collected from 80 pregnant women with IBD, including 90 samples from 58 women with CD and 34 samples from 22 women with UC. The mean age of participants was 30.52 ± 5.84 years, and the mean gestational age at enrollment was 21.69 ± 9.41 weeks. The samples were distributed across pregnancy trimesters, with 33 collected during the first, 49 during the second, and 42 during the third trimester. No statistically significant difference was found between patients with UC and those with CD in age, gestational week, disease duration, hemoglobin, CRP levels, ESR levels and disease activity at the beginning of pregnancy. Sample characteristics are elaborated in Table 1.

In the CD group, disease activity according to the CDAI was distributed as follows: 66 patients (73.33%) were in remission (CDAI < 150), 15 patients (16.66%) had mild to moderate disease (CDAI 150–220), and 9 patients (10%) had moderate to severe disease (CDAI 220–450).

In the UC group, disease activity based on the partial Mayo score was distributed as follows: 11 patients (32.23%) were in remission (partial Mayo 0–1), 17 patients (50%) had mild disease (partial Mayo 2–4), 4 patients (11.76%) had moderate disease (partial Mayo 5–6), and 2 patients (6.01%) had severe disease (partial Mayo 7–9).

Correlations between various inflammatory markers and clinical questionnaires: A strong linear correlation was demonstrated between chitinase and the partial Mayo score (r_p_ = 0.658, *p* < 0.001), while the linear correlation between chitinase and CDAI was weak to moderate (r_p_ = 0.304, *p* = 0.008). Calprotectin demonstrated a moderate-to-strong linear correlation with the Partial Mayo score and a moderate-to-strong non-linear correlation with the CDAI questionnaire. See Table 2.

Correlations between the various inflammatory markers: Significant positive correlations were observed among several inflammatory markers when examining all IBD patients, as described in Table 3. Serum CHI3L1 levels correlated with calprotectin (r_p_ = 0.366, *p* = 0.008) in a low-to-moderate correlation, suggesting its potential as an alternative inflammation marker. CHI3L1 also demonstrated a low-to-moderate correlation with ESR (r_p_ = 0.358, *p* = 0.001) and a moderate correlation with CRP (r_p_ = 0.478, *p* < 0.001). CRP exhibited a strong positive correlation with ESR (r_p_ = 0.614, *p* < 0.001), while CRP and calprotectin demonstrated a low-to-moderate non-linear correlation (r_s_ = 0.329, *p* = 0.008).

Correlation in IBD subgroups: When examining the population of patients with UC, a stronger linear correlation was observed between chitinase and calprotectin (r_p_ = 0.684, *p* = 0.007); see Table 4.

In patients with CD, the correlation between chitinase and CRP was moderate (r_p_ = 0.504, *p* < 0.001); between chitinase and CDAI, there was a moderate linear correlation, and the correlation with calprotectin was not statistically significant (r_p_ = 0.287, *p* = 0.080). See Table 5.

Regression models to predict active disease: In the multivariate regression model, elevated CHI3L1 levels (>56.6 ng/mL) were identified as a significant risk factor for active disease (OR 9.41, 95% CI 1.59–55.42), even after accounting for activity prior to pregnancy, age, CRP, hemoglobin and time from diagnosis (see Table 6). Post hoc analysis indicated that, with a sample size of 124, α = 0.05, and an observed odds ratio of 9.415, the study had a high statistical power of 0.92 to detect the observed effect.

## 4. Discussion

In this study, the serum levels of Chitinase 3-like-1 (CHI3L1) were measured in pregnant patients with IBD to characterize CHI3L1 levels in this population and to assess its potential as an inflammatory marker. Clinical monitoring of affected women is typically conducted through medical history, inflammatory markers such as CRP or stool calprotectin, intestinal ultrasound in selected sites, and clinical questionnaires in research settings. These methods have limitations due to the subjective nature of medical history and questionnaires, as well as the accessibility challenges of stool calprotectin and intestinal ultrasound and the influence of multiple factors on inflammatory markers such as CRP and ESR. One such factor is pregnancy, which inherently elevates inflammatory markers. A meta-analysis has questioned the reliability of CRP as an inflammatory marker for monitoring disease activity, even in non-pregnant IBD patients [20].

Our study found statistically significant evidence supporting the hypothesis that CHI3L1 can be a relevant marker for monitoring IBD activity in pregnancy. A significant correlation was observed between the Partial Mayo score or stool calprotectin and serum CHI3L1 levels in a small cohort of UC patients. When examining the correlation between CHI3L1 and CRP serum levels, a weak positive association was observed; however, this did not achieve statistical significance, potentially due to the limited sample size. These results indicate the need for further data collection and expansion of the UC patient group to substantiate this finding.

The CDAI is a commonly used index for assessing treatment response in clinical studies; however, its correlation with inflammatory status is not absolute [21]. During pregnancy, the reliability of the CDAI is further compromised, as several of its components, such as fatigue, abdominal discomfort, and altered bowel habits, can be influenced by normal physiological changes in pregnancy. This overlap diminishes the specificity and validity of the CDAI in assessing disease activity in pregnant patients with CD. We found a low-to-moderate positive correlation between CHI3L1 levels and CDAI, and a moderate positive correlation was observed between CHI3L1 and serum CRP levels.

Post hoc power analysis indicated that the study had adequate statistical power (0.92) to detect the overall observed effect size. Nonetheless, we acknowledge the modest size of subgroups; thus, while the overall findings are well-supported, the subgroup analyses remain exploratory and should be interpreted with caution.

The stronger correlations between serum CHI3L1 and inflammatory activity in UC compared with CD may reflect differences in epithelial involvement, mucosal injury, and innate immune activation, as suggested in earlier work demonstrating epithelial-driven upregulation of CHI3L1 in UC [15,22]. Other studies, however, describe CHI3L1 involvement in CD-related immune pathways and autoantigenicity [23], indicating that CHI3L1 biology may differ across IBD subtypes. Given our limited sample size, these findings should be interpreted with caution, and larger studies are needed to better understand subtype-specific CHI3L1 patterns.

Currently, serum CHI3L1 levels are not included in routine clinical panels. Studies had evaluated CHI3L1 levels in the generally healthy, non-pregnant population. While data on healthy pregnant women are limited, studies on pregnancies with various pathologies allow for some inference regarding CHI3L1 levels in healthy pregnant women. However, no studies have yet investigated CHI3L1 levels in pregnant women with IBD. As this is a pioneering study examining CHI3L1 levels in this unique population, an attempt was made to define a threshold CHI3L1 level indicative of inflammation (based on currently accepted parameters such as CRP and ESR). However, this threshold could not be determined in the present study due to the small sample size, which precluded a reliable ROC curve analysis.

This is a preliminary study and, as such, has several limitations. It is a single-center study with small study groups and multiple samples from the same patients, necessitating an increase in participant numbers in each subgroup to enhance the statistical power of the conclusions. Serum CHI3L1 levels were measured using a manual ELISA assay, which is susceptible to bias due to variations in test performance. The influence of medication on inflammation and CHI3L1 levels could not be analyzed despite data collection, as categorizing patients by treatment resulted in subgroups too small for meaningful comparisons.

However, adjusting the model for medication exposure, including a specific check for thiopurines, did not change the odds ratio. Thus, although treatment effects may introduce bias, they did not influence the observed association between CHI3L1 and active disease in our cohort. Future research should prioritize assessing the impact of specific treatments on CHI3L1 levels.

All IBD patients in this study were recruited from the IBD MOM clinic, a specialized multidisciplinary clinic. As a result, most study participants were in remission at conception, with only a few experiencing active diseases during pregnancy. Additionally, it remains unknown whether women who declined participation in the study differed in characteristics from those who agreed, as no data were collected on this subgroup. A comprehensive understanding of serum CHI3L1 levels in pregnant IBD patients requires data collection from untreated patients, those not under clinical follow-up, and those with highly active diseases. Recruiting such a diverse cohort is technically challenging and necessitates a long-term study beyond the scope of the current research.

It should also be noted that pregnancy-related physiological factors may influence serum CHI3L1 levels independently of inflammatory activity. Variations in gestational age, physiological hemodilution, and metabolic or hormonal adaptations throughout pregnancy could potentially affect CHI3L1 concentrations and therefore represent important confounders when interpreting this biomarker in pregnant patients with IBD. Further studies are needed to clarify the extent of these effects.

Furthermore, CHI3L1 levels in intestinal tissue rather than serum may better reflect disease activity [24]. Similar findings regarding calprotectin exist in the literature. Ongoing research in our laboratory aims to assess CHI3L1 levels in intestinal tissue from IBD patients. Although unpublished, preliminary data indicate a positive correlation between tissue CHI3L1 levels and intestinal disease status. These findings and other studies suggest the potential for further investigation and expansion.

The next research phase following this study will focus on increasing the sample size to validate the observed correlations. Additionally, future research should explore the relationship between CHI3L1 and pregnancy outcomes, evaluate other serum proteins as potential inflammatory markers in this unique population, and examine CHI3L1 correlations with currently accepted inflammatory markers.

In conclusion, this study strengthens the hypothesis that CHI3L1 has the potential to be a reliable inflammatory marker in pregnant IBD patients, both CD and UC, likely with greater relevance for the UC subgroup. Since pregnancy is a medical condition in which invasive procedures are not recommended, personalized treatment for pregnant IBD patients can be tailored according to CHI3L1 levels.

## Figures and Tables

**Table 1:** Characteristics of study participants by study group.

	All (N = 124)	UC (N = 34)	CD (N = 90)	*p* Value
Age	30.52 ± 5.83	29.79 ± 6.26	30.79 ± 5.67	0.399
Gestational age at enrollment	21 (14–32)	20 (13.5–30)	22 (13.75–22)	0.666
Erythrocyte Sedimentation Rate (mm/hr)	28.67 ± 19.50	23.79 ± 13.67	30.57 ± 21.13	0.119
C-reactive protein (mg/dL)	1.32 ± 2.12	1.03 ± 0.99	1.43 ± 2.42	0.347
Eosinophils (K/microL)	0.27 ± 0.66	0.41 ± 0.71	0.22 ± 0.63	0.158
Hemoglobin (g/dL)	11.74 ± 1.11	11.81 ± 1.14	11.71 ± 1.11	0.637
Calprotectin (mcg/gr)	857 ± 1024	1229 ± 1167	719 ± 942	0.079
CHI3L1 (ng/mL)	38.32 ± 35.35	33.09 ± 21	40.51 ± 39.78	0.329
Disease duration (years)	6 (2–12)	1.5 (1–8)	6.5 (4–14)	<0.001
*Medications*				
vedolizumab	4 (3.2)	0 (0)	4 (4.4)	0.574
thiopurines	38 (30.6)	5 (14.7)	33 (36.7)	0.018
5-ASA	42 (33.9)	28 (82.4)	14 (15.6)	<0.001
Steroids	16 (12.9)	4 (11.8)	12 (13.3)	0.999
infliximab	21 (16.9)	8 (23.5)	13 (14.4)	0.229
Humira adalimumab	19 (15.3)	0 (0)	19 (21.1)	0.004
Disease activity: active vs. remission	39 (31.5)	15 (44.1)	24 (26.7)	0.062
Active disease pre-pregnancy	36 (29.3)	14 (41.2)	22 (24.7)	0.073

**Table 2:** Correlations between inflammatory markers and validated clinical questionnaire.

		Partial MAYO (av. 3d)	CDAI
ESR (mm/hr)	Correlation coefficient	−0.017 ^&^	0.297 ^$^
	*p* value	0.931	0.011
CRP (mg/dL)	Correlation coefficient	0.216 ^&^	0.416 ^$^
	*p* value	0.220	<0.001
Calprotectin (mcg/gr)	Correlation coefficient	0.580 ^$^	0.535 ^&^
	*p* value	0.015	<0.001
CHI3L1 (ng/mL)	Correlation coefficient	0.658 ^$^	0.297 ^$^
	*p* value	<0.001	0.010

^$^ Pearson’s r. ^&^ Spearman’s rho.

**Table 3:** Correlation matrix between inflammatory markers.

		CHI3L1 (ng/mL)	Calprotectin (mcg/gr)	CRP (mg/dL)
ESR (mm/hr)	Correlation coefficient	0.358 ^$^	0.245 ^&^	0.614 ^$^
	*p* value	<0.001	0.086	<0.001
CRP (mg/dL)	Correlation coefficient	0.478 ^$^	0.329 ^&^	
	*p* value	<0.001	0.008	
calprotectin (mcg/gr)	Correlation coefficient	0.366 ^$^		
	*p* value	0.008		

^$^ Pearson’s r. ^&^ Spearman’s rho.

**Table 4:** Correlation matrix between inflammatory markers and partial MAYO score in the UC group.

		CRP (mg/dL)	Calprotectin (mcg/gr)	CHI3L1 (ng/mL)
calprotectin (mcg/gr)	r_p_	0.055		
	*p* value	0.833		
CHI3L1 (ng/mL)	r_p_	0.153	0.684	
	*p* value	0.411	0.007	
Partial MAYO score	r_p_	0.137	0.580	0.658
	*p* value	0.440	0.015	<0.001

**Table 5:** Correlation matrix between inflammatory markers and CDAI score in the CD group.

		Calprotectin (mcg/gr)	CHI3L1 (ng/mL)	CDAI
CRP (mg/dL)	Correlation coefficient	0.306 ^$^	0.504 ^$^	0.416 ^$^
	*p* value	0.039	<0.001	<0.001
Calprotectin (mcg/gr)	Correlation coefficient		0.287 ^$^	0.535 ^&^
	*p* value		0.080	<0.001
CHI3L1 (ng/mL)	Correlation coefficient			0.297 ^$^
	*p* value			0.010

^$^ Pearson’s r. ^&^ Spearman’s rho.

**Table 6:** Multivariate logistic regression model for the prediction of active disease.

Variable	OR	95% CI	*p* Value
Elevated CHI3L1 (>56.6 ng/mL)	9.41	1.59–55.42	0.013
Pre-pregnancy activity	5.78	1.25–26.8	0.025
Age	0.87	0.75–1.00	0.060
Hemoglobin (g/dL)	0.59	0.28–1.24	0.166
CRP (mg/dL)	1.02	0.68–1.55	0.915
Time from diagnosis	1.04	0.91–1.18	0.603
Active treatment	0.57	0.09–3.43	0.543

## Data Availability

Data is unavailable due to privacy and ethical restrictions.
